# Integrative Transcriptomic and Single‐Cell Analyses Identify ATP1A1 as a Prognostic and Immune‐Associated Factor in Esophageal Cancer

**DOI:** 10.1155/ijog/1461834

**Published:** 2026-06-03

**Authors:** Huishen Yan, Liang He

**Affiliations:** ^1^ Department of Medical Science, Yangzhou Polytechnic University, Yangzhou, China; ^2^ Department of Neurosurgery, Northern Jiangsu People’s Hospital Affiliated to Yangzhou University, Yangzhou, China, yzu.edu.cn

**Keywords:** ATP1A1, drug response, esophageal cancer, mitochondrial stress, prognosis, single-cell transcriptomics, tumor microenvironment

## Abstract

**Background:**

Esophageal cancer is a highly aggressive malignancy with poor prognosis and limited molecular markers for effective risk stratification and therapeutic guidance. Mitochondrial stress–related pathways are increasingly recognized as important regulators of tumor progression and immune modulation; however, their clinical relevance in esophageal cancer remains insufficiently characterized.

**Methods:**

Transcriptomic and clinical data of esophageal cancer were obtained from public databases. A predefined mitochondrial stress–related gene set was analyzed using LASSO Cox regression to construct a prognostic model. Survival analysis, functional enrichment, immune regulatory profiling, and immune cell infiltration analyses were subsequently performed. Single‐cell RNA sequencing data from the GSE160269 cohort were used to localize gene expression within the tumor microenvironment. In addition, pan‐cancer drug sensitivity analyses were conducted using pharmacogenomic datasets.

**Results:**

ATP1A1 was identified as the only LASSO‐selected gene demonstrating consistent prognostic significance, with higher expression associated with improved survival outcomes. Functional analyses indicated that ATP1A1 expression was mainly associated with metabolic pathways and negatively correlated with epithelial–mesenchymal transition, invasion, and quiescence states. Immune analyses showed that ATP1A1 expression was associated with heterogeneous immunomodulatory patterns, differences in immune cell infiltration, and reduced activity across several steps of the cancer immunity cycle. Single‐cell analysis demonstrated that ATP1A1 expression was not only preferentially enriched in malignant epithelial cells but also detectable in stromal and immune cell populations. Drug sensitivity analyses suggested that ATP1A1 expression was associated with differential responses to multiple therapeutic agents. Experimental validation further showed that ATP1A1 knockdown altered tumor cell behavior and increased inflammatory gene expression in esophageal cancer cells.

**Conclusions:**

ATP1A1 represents a prognostically relevant factor linked to tumor biological characteristics, immune microenvironment features, and therapeutic response heterogeneity in esophageal cancer.

## 1. Introduction

Esophageal cancer represents a highly lethal malignancy with persistently poor clinical outcomes worldwide [1, 2]. Despite advances in surgical techniques, chemoradiotherapy, and immunotherapy, the overall survival of patients with esophageal cancer remains poor [3], largely due to pronounced intertumoral heterogeneity and the absence of robust molecular markers that can reliably stratify prognosis and inform therapeutic decision‐making [4, 5]. Thus, identifying clinically relevant molecular features that link tumor biological states with the immune microenvironment remains an important unmet need in esophageal cancer research.

Cellular adaptation to mitochondrial stress has emerged as an important determinant of tumor biological behavior [6, 7]. Beyond their classical functions in energy production, mitochondria act as signaling hubs that coordinate apoptotic regulation, redox homeostasis, membrane integrity, and inflammatory signaling [5, 8]. Recent studies suggest that mitochondrial stress–associated pathways may contribute to tumor progression and therapy response through coordinated effects on both cancer cells and the tumor microenvironment [9, 10]. However, their clinical relevance and transcriptomic associations in esophageal cancer remain insufficiently defined.

ATP1A1, encoding the *α*1 subunit of the Na^+^/K^+^‐ATPase, has traditionally been regarded as a membrane ion transporter essential for maintaining cellular electrochemical gradients [11]. Emerging studies, however, have revealed that ATP1A1 functions extend beyond ion homeostasis, participating in signal transduction, redox regulation, and stress adaptation pathways that are highly relevant to cancer biology [12, 13]. Altered ATP1A1 expression has been reported in several malignancies and has been associated with tumor progression and drug response, yet its prognostic significance, immune associations, and cellular distribution in esophageal cancer remain unclear [14].

Recent advances in integrative transcriptomic analyses and single‐cell technologies provide an unprecedented opportunity to dissect gene function across multiple biological scales [15, 16]. Bulk RNA sequencing enables the evaluation of prognostic relevance and pathway‐level associations, while immune deconvolution algorithms allow the interrogation of tumor–immune interactions. In parallel, single‐cell RNA sequencing can localize gene expression to specific cellular compartments within the tumor microenvironment, thereby resolving ambiguities inherent to bulk‐level analyses [17, 18]. Integration of these approaches with pharmacogenomic resources further enables exploratory assessment of potential therapeutic associations.

Here, we systematically evaluated mitochondrial stress–associated genes in esophageal cancer using a multilayer analytical framework and identified ATP1A1 as a prognostically relevant candidate through LASSO Cox modeling. We subsequently characterized ATP1A1 expression patterns, survival associations, transcriptomic pathway correlations, immune‐related features, and immune infiltration patterns. Single‐cell RNA sequencing was further used to determine the cellular localization of ATP1A1 within the tumor microenvironment, and pharmacogenomic datasets were analyzed to explore potential drug response associations. Collectively, this study was designed to provide an integrative characterization of ATP1A1 across prognostic, transcriptomic, immune, and cellular contexts. Rather than establishing causal biological mechanisms, our findings should be interpreted as association‐based observations that may inform future functional investigations.

## 2. Methods

### 2.1. Data Acquisition and Preprocessing

RNA sequencing data together with matched clinical annotations were retrieved from The Cancer Genome Atlas (TCGA) database [19]. Expression profiles generated using the STAR workflow were transformed into transcript per million (TPM) format, followed by *l* *o* *g*2(*T* *P* *M* + 1) normalization to reduce data variability [20]. Samples with duplicated records or missing survival information were excluded. Only cases with complete expression and survival data were retained for downstream analyses, resulting in a final cohort of 163 patients.

### 2.2. Feature Selection Using LASSO Cox Regression

To screen prognostically relevant genes from the predefined mitochondrial stress–related gene panel, LASSO Cox regression analysis was conducted using the glmnet R package [21]. A 10‐fold cross‐validation procedure was applied to determine the optimal penalty parameter (*λ*). Gene coefficient trajectories and model deviance across different *λ* values were examined. Genes maintaining nonzero coefficients at the selected *λ* value were retained for subsequent analyses.

### 2.3. Construction of the Prognostic Risk Model

Selected genes were incorporated into a multivariable Cox proportional hazards model to construct a prognostic signature. A stepwise model optimization strategy based on the Akaike information criterion (AIC) was performed using the survival package. Individual risk scores were calculated as the weighted sum of gene expression levels according to their corresponding regression coefficients.

### 2.4. Survival Analysis and Model Evaluation

Patients were stratified into high‐ and low‐risk groups according to the median risk score. Kaplan–Meier survival curves were generated to compare overall survival between groups, and statistical significance was evaluated using the log‐rank test. Hazard ratios (HRs) together with 95% confidence intervals (CIs) were estimated using Cox regression analysis. Time‐dependent receiver operating characteristic (ROC) curves were generated to assess predictive performance at multiple follow‐up time points, and the area under the curve (AUC) was calculated to quantify model discrimination ability [22].

### 2.5. Gene Set Enrichment Analysis (GSEA) and Functional State Association Analysis

Patients were categorized according to ATP1A1 expression levels, with the top 30% classified as the high‐expression group and the bottom 30% as the low‐expression group. Differential expression analysis between these two groups was conducted using the limma R package [23]. Genes were ranked according to differential expression statistics to generate a preranked gene list for gene set enrichment analysis (GSEA) [24], which was performed using the clusterProfiler R package [25] based on KEGG pathway annotations [26]. Normalized enrichment scores (NES) were calculated to quantify pathway enrichment, and multiple testing correction was applied to control statistical significance. To further investigate functional characteristics associated with ATP1A1, CancerSEA‐defined gene signatures [27] were collected and evaluated using the GSVA R package [28] with the *z*‐score method. Pearson’s correlation analysis was subsequently conducted to assess relationships between ATP1A1 expression levels and tumor functional state scores.

### 2.6. Association Between ATP1A1 Expression and Immunomodulatory Features

Immune‐related molecular features were evaluated using datasets obtained from the TISIDB database [29], which integrates multiple resources on tumor–immune system interactions, immunostimulatory genes, immunoinhibitory genes, chemokines, and human leukocyte antigen (HLA) genes. Patients with esophageal cancer were divided into high‐ and low‐expression groups according to ATP1A1 levels. Differences in immune‐related gene expression between the two groups were examined. Mean expression values of each immune‐related gene in the two groups were visualized using heatmaps. To further characterize the regulatory context of immunomodulatory molecules, samples were stratified into ATP1A1 expression quartiles (Q1–Q4). For each quartile, median mRNA expression levels of immunomodulators, correlations between gene expression and DNA methylation (*β* values), and somatic copy number alteration (SCNA) patterns, including amplification and deletion frequencies, were analyzed following the framework described by Thorsson et al. [30]. Immune response–related scores and genome status scores were similarly summarized across ATP1A1 expression quartiles, and heatmaps were generated using the pheatmap R package with row‐wise standardization.

### 2.7. Association Between ATP1A1 Expression and Immune Cell Infiltration

Immune cell infiltration profiles of TCGA esophageal cancer samples were retrieved from the TIMER2.0 database [31], which integrates multiple computational methods for immune deconvolution. Spearman’s correlation analysis was conducted to explore relationships between ATP1A1 expression and the estimated abundance of infiltrating immune cell populations across different algorithms. Correlation coefficients with *p* < 0.05 were considered statistically significant and were displayed using heatmaps. To further compare immune infiltration patterns between ATP1A1 high‐ and low‐expression groups, patients were dichotomized according to the median ATP1A1 expression level. Differences in immune cell abundance estimated by seven independent algorithms were evaluated using the Wilcoxon rank‐sum test. Immune cell types showing significant differences were visualized using heatmaps, with samples ordered according to increasing ATP1A1 expression. In addition, cancer immunity cycle activity was assessed using tracking tumor immunophenotype (TIP) scores. Spearman’s correlation analysis was also conducted to evaluate associations between ATP1A1 expression and individual TIP scores, as well as the correlations among different TIP steps. Visualization was performed using the linkET R package.

### 2.8. Association Between ATP1A1 Expression and Drug Sensitivity in Pan‐Cancer

Drug response data were obtained from the Genomics of Drug Sensitivity in Cancer (GDSC) [32] and Cancer Therapeutics Response Portal (CTRP) databases [33]. For each dataset, associations between ATP1A1 mRNA expression and drug response values were calculated across pan‐cancer cell line panels. Spearman’s correlation analysis was performed to evaluate relationships between gene expression and drug sensitivity. Multiple testing correction was conducted using the false discovery rate (FDR) approach. Drugs with FDR ≤ 0.05 were considered statistically significant, and the top 30 compounds ranked by statistical significance were visualized. Positive correlations indicate that elevated ATP1A1 expression is associated with increased drug resistance, whereas negative correlations indicate enhanced drug sensitivity.

### 2.9. Single‐Cell RNA Sequencing Analysis of ATP1A1 Expression in Esophageal Cancer

Single‐cell transcriptomic data of esophageal cancer were retrieved from the publicly available dataset GSE160269 [34], which profiles primary esophageal cancer samples using the 10x Genomics platform. Publicly available expression matrices were processed using standard single‐cell analysis procedures, including quality control, normalization, scaling, and dimensionality reduction. Uniform manifold approximation and projection (UMAP) was applied to visualize the cellular landscape [35]. To reduce the impact of dropout events, ATP1A1 expression was visualized using the Nebulosa R package [36]. Cell clusters were annotated according to canonical marker genes to define major cell types within the tumor microenvironment. Cells were further classified into ATP1A1‐positive and ATP1A1‐negative groups based on detectable expression, and the proportional distribution of annotated cell types was calculated for both groups. Differences in ATP1A1 expression across cell populations were evaluated using the Kruskal–Wallis rank‐sum test and visualized using violin plots, boxplots, and bar plots.

### 2.10. Cell Lines and Cell Culture

The human normal esophageal epithelial cell line Het‐1A (RRID: CVCL_3702), the esophageal squamous cell carcinoma cell lines KYSE150 (RRID: CVCL_1348) and Eca109 (RRID: CVCL_6898), and the human embryonic kidney cell line HEK293 (RRID: CVCL_0045) were included in this study. Cells were maintained in Dulbecco’s modified Eagle medium (DMEM) (Gibco, Cat. No. 11965092) supplemented with 10% fetal bovine serum (FBS) (Gibco, Cat. No. 10099141C) and 1% penicillin–streptomycin (Gibco, Cat. No. 15140122) at 37°C in a humidified atmosphere containing 5% CO_2_. All cell lines were routinely screened and confirmed to be free of mycoplasma contamination prior to experimentation. All cell lines were routinely tested for mycoplasma contamination and were obtained from commercial sources with authentication information provided by the vendors.

### 2.11. Quantitative Real‐Time PCR

Total RNA was extracted from cultured cells using TRIzol reagent (Invitrogen, Thermo Fisher Scientific, Cat. No. 15596026) according to the manufacturer’s protocol. RNA concentration and purity were determined by measuring absorbance at 260 and 280 nm using a NanoDrop One spectrophotometer (Thermo Scientific, United States). Complementary DNA (cDNA) was synthesized from total RNA using the PrimeScript RT reagent kit with gDNA Eraser (Takara, Cat. No. RR047A) following the supplier’s instructions. Quantitative real‐time PCR (qPCR) was performed using TB Green Premix Ex Taq II (Takara, Cat. No. RR820A) on a QuantStudio 5 Real‐Time PCR system (Applied Biosystems, United States). Each reaction was prepared in a 20‐*μ*L volume containing TB Green Premix, gene‐specific primers, cDNA template, and nuclease‐free water. The amplification protocol included an initial denaturation at 95°C for 30 s, followed by 40 cycles of 95°C for 5 s and 60°C for 30 s. Melt curve analysis was conducted to verify amplification specificity. Relative gene expression levels were calculated using the 2^−^ *ΔΔ*Ct method with GAPDH as the internal reference gene. All experiments were performed in triplicate, and each assay was independently repeated at least three times. The primer sequences used in this study were as follows:

− GAPDH‐F: GACAGTCAGCCGCATCTTCT
− GAPDH‐R: GCGCCCAATACGACCAAATC
− IL6‐F: GTCCAGTTGCCTTCTCCCTGG
− IL6‐R: CCCATGCTACATTTGCCGAAG
− IL1B‐F: AACCTCTTCGAGGCACAAGG
− IL1B‐R: AGATTCGTAGCTGGATGCCG
− TNF‐F: AAAACAACCCTCAGACGCCA
− TNF‐R: TCCTTTCCAGGGGAGAGAGG
− CXCL8‐F: AGAGAGCTCTGTCTGGACCC
− CXCL8‐R: TTCTCAGCCCTCTTCAAAAACT
− PTGS2‐F: AGGCTTCCATTGACCAGAGC
− PTGS2‐R: TCCACAGCATCGATGTCACC
− ATP1A1‐F: CTCTGATTCTCCAGCGACAGG
− ATP1A1‐R: AACAGCTGCAGGCTCATACTT

### 2.12. Stable ATP1A1 Knockdown and Validation

Stable ATP1A1 knockdown was established using a lentiviral shRNA (short hairpin RNA) approach. shRNA constructs targeting ATP1A1 and a nontargeting control (shNC) were inserted into the pLKO.1 lentiviral vector (Addgene, Cat. No. 8453). Lentiviral particles were produced by cotransfecting HEK293 cells with the shRNA constructs together with the packaging plasmids psPAX2 (Addgene, Cat. No. 12260) and pMD2.G (Addgene, Cat. No. 12259) using Lipofectamine 3000 (Invitrogen, Thermo Fisher Scientific, Cat. No. L3000015) according to the manufacturer’s protocol. After 48–72 h, viral supernatants were harvested, passed through a 0.45‐*μ*m membrane filter (Millipore, Cat. No. SLHV033RS), and used to infect KYSE150 and Eca109 cells in the presence of polybrene (Sigma‐Aldrich, Cat. No. TR‐1003). Following transduction, cells were maintained in medium supplemented with puromycin (Sigma‐Aldrich, Cat. No. P8833) for approximately 7–10 days to generate stable knockdown cell lines. Knockdown efficiency of ATP1A1 was subsequently confirmed by quantitative real‐time PCR. shRNA sequences are provided in Table S1.

### 2.13. Cell Counting Kit‐8 (CCK‐8) Assay

Cell viability was evaluated using CCK‐8 (Dojindo Laboratories, Japan, Cat. No. CK04) following the manufacturer’s instructions. KYSE150 and Eca109 cells with stable ATP1A1 knockdown or control cells (shNC) were seeded into 96‐well plates at a density of approximately 2 × 10^4^ cells per well in 100‐*μ*L complete culture medium. At the indicated time points (0, 24, 48, 72, and 96 h), 10 *μ*L of CCK‐8 reagent was added to each well, followed by incubation at 37°C for 1–2 h. Absorbance was then measured at 450 nm using a microplate reader (BioTek Synergy HTX, Agilent Technologies, United States) to determine cell viability. Each experimental condition included three technical replicates, and all experiments were independently repeated at least three times.

### 2.14. Statistical Analysis

All statistical analyses were conducted using R software (Version 4.0.3) and GraphPad Prism 9. Continuous variables between two groups were compared using the Wilcoxon rank‐sum test, while comparisons among multiple groups were performed using the Kruskal–Wallis test when appropriate. Survival differences were evaluated using Kaplan–Meier analysis with the log‐rank test. HRs and 95% CIs were calculated using Cox proportional hazards regression models. Correlation analyses were performed using Pearson’s or Spearman’s methods as appropriate. For in vitro experiments, data are presented as mean ± SD from at least three independent experiments. Differences between two groups were analyzed using Student’s *t*‐test, while multiple‐group comparisons were assessed using one‐way ANOVA. Two‐way ANOVA was used for CCK‐8 assays. A two‐sided *p* < 0.05 was considered statistically significant.

## 3. Results

### 3.1. LASSO Screening Identifies Prognostic Genes and a Survival Risk Pattern

**Figure 1:**
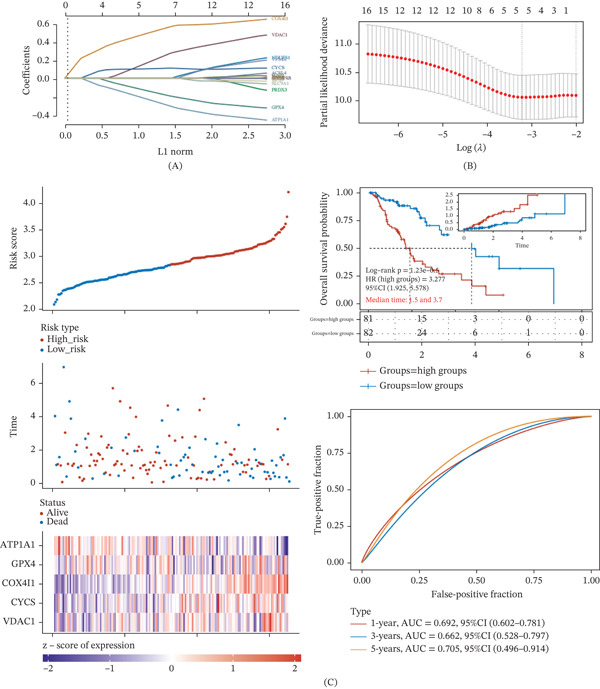
Identification of prognostic genes and construction of a LASSO Cox risk model in esophageal cancer. (A) Coefficient profiles of mitochondrial stress–related genes across log(*λ*) values in the LASSO Cox regression model, showing shrinkage of regression coefficients with increasing penalization. (B) Tenfold cross‐validation used to determine the optimal penalty parameter (*λ*) based on minimum partial likelihood deviance. (C) Distribution of risk scores, survival status, survival time, and expression patterns of the five selected genes in the TCGA esophageal cancer cohort. Samples are arranged according to increasing risk score.

LASSO Cox regression was performed on the predefined mitochondrial stress–related gene set to identify key prognostic candidates, yielding five genes with nonzero coefficients at the optimal penalty parameter (*λ*) selected through 10‐fold cross‐validation, including ATP1A1, GPX4, COX4I1, CYCS, and VDAC1 (Figure 1A,B). A multivariate Cox regression model was then established to calculate individualized risk scores for each patient. When samples were ordered according to increasing risk scores, patients with higher scores exhibited increased mortality risk, accompanied by distinct expression patterns of the five selected genes across the risk groups, supporting effective patient stratification (Figure 1C). Based on the median risk score, patients were categorized into high‐ and low‐risk groups. Kaplan–Meier survival analysis demonstrated significantly poorer overall survival in the high‐risk group compared with the low‐risk group (log‐rank *p* = 1.23 × 10^−5^, HR = 3.277, 95*%*CI = 1.925–5.578), with median survival times of 1.5 and 3.7 years, respectively (Figure 1C). Time‐dependent ROC analysis further confirmed the prognostic performance of the model, with AUC values of 0.692, 0.662, and 0.705 at 1, 3, and 5 years, respectively (Figure 1C).

### 3.2. ATP1A1 Is the Only LASSO‐Selected Candidate With Prognostic Relevance in Esophageal Cancer

**Figure 2:**
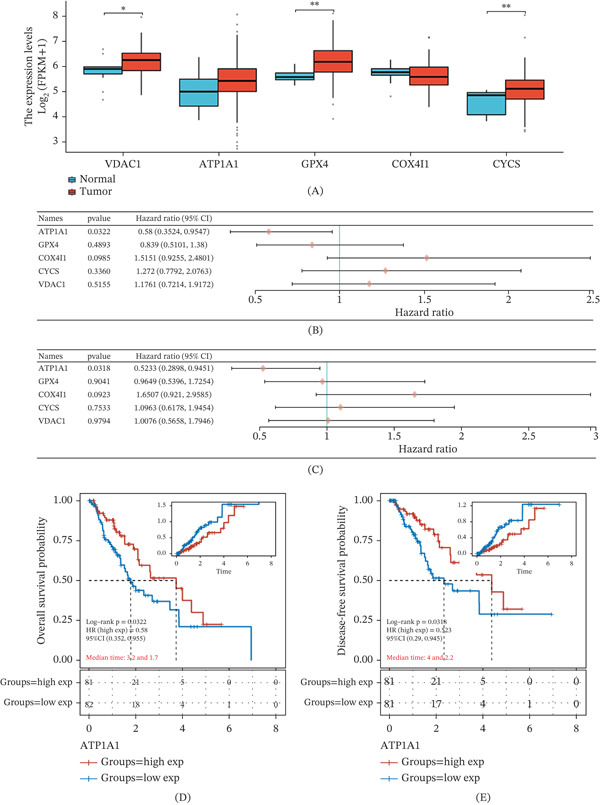
Identification of ATP1A1 as the only prognostically relevant LASSO candidate in esophageal cancer. (A) Comparison of mRNA expression of five LASSO‐selected genes between tumor and normal tissues in TCGA. (B) Univariate Cox regression analysis of overall survival for candidate genes. (C) Univariate Cox regression analysis of disease‐specific survival. (D) Kaplan–Meier overall survival curves stratified by ATP1A1 expression. (E) Kaplan–Meier disease‐specific survival curves stratified by ATP1A1 expression.

Expression levels of the five LASSO‐selected genes were initially compared between esophageal cancer and normal tissues. Among these genes, VDAC1, GPX4, and CYCS were significantly upregulated in tumor tissues, whereas ATP1A1 and COX4I1 showed no significant differences between tumor and normal samples. Notably, ATP1A1 displayed a tendency toward increased expression in tumors (Figure 2A). The prognostic significance of these genes was further examined using univariate Cox regression analysis. Among them, ATP1A1 was significantly associated with overall survival, acting as a protective factor (*H* *R* = 0.58, 95*%* *C* *I* = 0.352–0.955, *p* = 0.032), while the remaining genes showed no significant associations with survival outcomes (Figure 2B). A comparable trend was also observed for disease‐specific survival, with ATP1A1 retaining statistical significance (*H* *R* = 0.523, 95*%* *C* *I* = 0.2898–0.9451, *p* = 0.0318), further supporting its prognostic relevance (Figure 2C). Kaplan–Meier analysis further demonstrated that patients with higher ATP1A1 expression experienced improved overall survival compared with those with lower expression (log‐rank *p* = 0.032, *H* *R* = 0.58, 95*%* *C* *I* = 0.352–0.955), with median survival times of 3.2 and 1.7 years, respectively (Figure 2D). Similar survival advantages were observed for disease‐specific survival (log‐rank *p* = 0.0318, *H* *R* = 0.5239, 95*%* *C* *I* = 0.29–0.945), with median survival times of 4.0 and 2.2 years, respectively (Figure 2E). Taken together, these results indicate that ATP1A1 is the only LASSO‐selected gene demonstrating consistent prognostic value in esophageal cancer and was therefore selected for subsequent analyses.

### 3.3. Transcriptional Programs and Functional States Associated With ATP1A1 Expression

**Figure 3:**
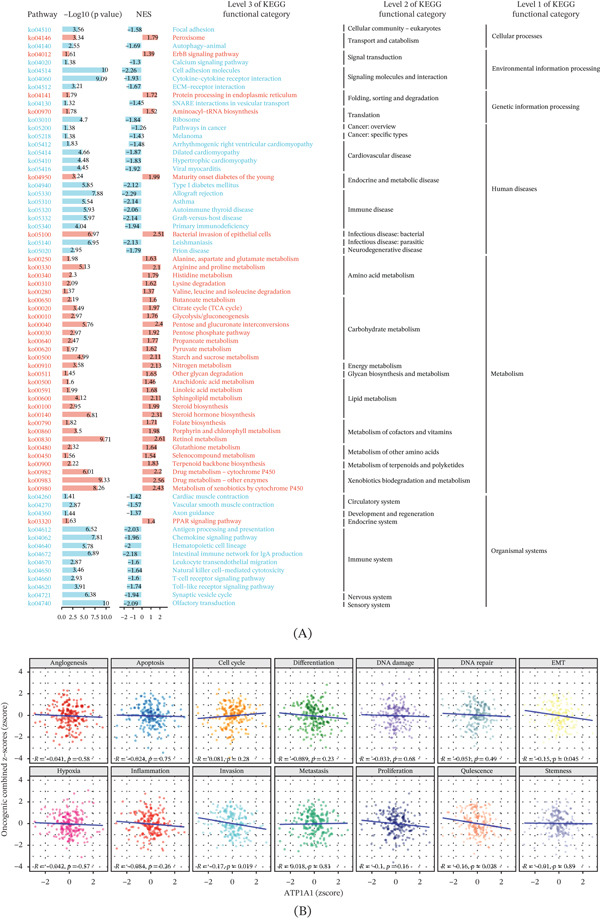
Functional enrichment landscape and tumor functional states associated with ATP1A1 expression. (A) Gene set enrichment analysis comparing ATP1A1 high‐ and low‐expression groups using KEGG pathways. Positive normalized enrichment scores indicate pathways enriched in ATP1A1 high‐expression samples, whereas negative scores indicate pathways enriched in low‐expression samples. (B) Correlation analysis between ATP1A1 expression and CancerSEA‐defined tumor cell functional states. Correlation coefficients indicate the strength and direction of associations.

To explore the biological programs associated with ATP1A1 expression in esophageal cancer, GSEA was conducted by comparing samples stratified by ATP1A1 expression levels. The enrichment landscape revealed bidirectional pathway alterations across multiple biological categories. Pathways with positive NES were mainly involved in metabolic processes, including amino acid metabolism, glycolysis/gluconeogenesis, pentose phosphate pathway, pyruvate metabolism, starch and sucrose metabolism, nitrogen metabolism, arachidonic acid metabolism, linoleic acid metabolism, sphingolipid metabolism, steroid hormone biosynthesis, folate biosynthesis, porphyrin and chlorophyll metabolism, retinol metabolism, glutathione metabolism, and drug metabolism mediated by cytochrome P450, as well as xenobiotic metabolism through cytochrome P450. In contrast, pathways with negative NES were primarily associated with cell adhesion, ECM–receptor interaction, cytokine–cytokine receptor interaction, antigen processing and presentation, chemokine signaling pathway, hematopoietic cell lineage, intestinal immune network for IgA production, leukocyte transendothelial migration, natural killer (NK) cell–mediated cytotoxicity, T‐cell receptor (TCR) signaling pathway, Toll‐like receptor signaling pathway, and synaptic vesicle cycle (Figure 3A). To further characterize the functional states associated with ATP1A1, correlations between ATP1A1 expression and CancerSEA‐defined tumor cell functional states were analyzed. Most functional states showed weak correlations with ATP1A1 expression, including angiogenesis, apoptosis, cell cycle, differentiation, DNA damage, DNA repair, hypoxia, inflammation, metastasis, proliferation, and stemness. In contrast, ATP1A1 expression was negatively correlated with epithelial–mesenchymal transition (EMT) (*R* = −0.15, *p* = 0.045), invasion (*R* = −0.17, *p* = 0.019), and quiescence (*R* = −0.16, *p* = 0.028), suggesting that higher ATP1A1 expression may be linked to reduced invasive, mesenchymal, and quiescent cellular phenotypes in esophageal cancer (Figure 3B).

### 3.4. ATP1A1 Expression Is Associated With Immunomodulatory Profiles and Immune‐Related Genomic States in Esophageal Cancer

**Figure 4:**
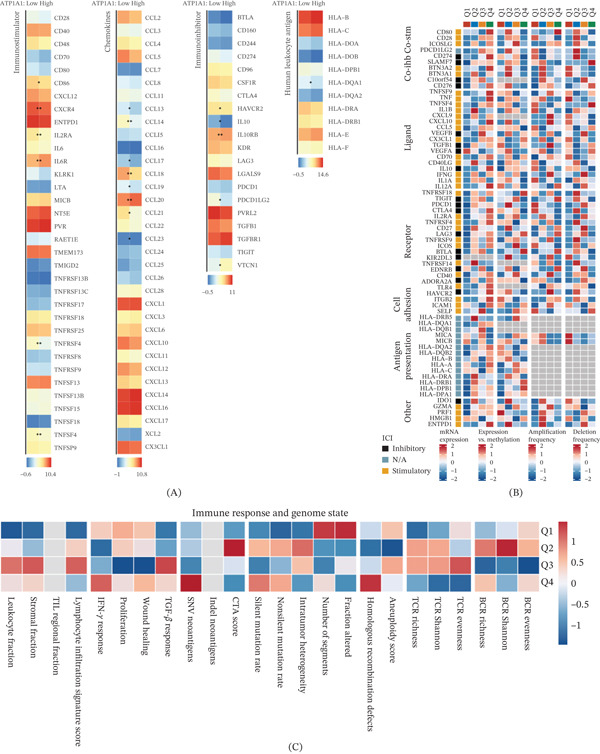
Association between ATP1A1 expression and immunomodulatory and genomic features in esophageal cancer. (A) Heatmap showing differential expression of immunostimulatory genes, immunoinhibitory genes, chemokines, and HLA genes between ATP1A1 high‐ and low‐expression tumors. (B) Multiomics landscape of immunomodulatory molecules across ATP1A1 expression quartiles, including mRNA expression patterns, DNA methylation correlations, and somatic copy number alteration frequencies. (C) Distribution of immune‐related and genome instability–related scores across ATP1A1 expression groups.

To investigate the immunological features associated with ATP1A1 expression in esophageal cancer, immune‐related gene expression profiles were analyzed between ATP1A1 low‐ and high‐expression groups. Broad but heterogeneous alterations were observed across multiple categories of immune‐related molecules, including immunostimulators, chemokines, immunoinhibitors, and HLA genes. Rather than showing a uniform increase or decrease, ATP1A1 high‐expression tumors exhibited selective upregulation of certain immune‐related genes together with reduced expression of others, indicating a complex immunomodulatory pattern linked to ATP1A1 status (Figure 4A). Further characterization of the regulatory landscape underlying these differences was performed by stratifying samples according to ATP1A1 expression quartiles and integrating multilayer immunogenomic features. Distinct quartile‐dependent patterns were observed in the mRNA expression of immunomodulators, their correlations with DNA methylation, and SCNA frequencies, including both amplification and deletion events, suggesting that ATP1A1‐associated immune variation may be linked to coordinated transcriptional, epigenetic, and genomic regulatory changes rather than a single linear trend (Figure 4B). In addition, immune response and genome stability features also varied across ATP1A1 quartiles. Features related to leukocyte and stromal fractions, lymphocyte infiltration signature, IFN‐*γ* response, proliferation, wound healing, TGF‐*β* response, neoantigen‐related indices, mutation rates, intratumor heterogeneity, copy number alteration burden, homologous recombination defects, aneuploidy, and TCR/BCR repertoire characteristics showed distinct quartile‐specific distributions, further supporting an association between ATP1A1 expression and broad remodeling of the immune and genomic landscape in esophageal cancer (Figure 4C).

### 3.5. Immune Infiltration Patterns and Cancer Immunity Cycle Features Linked to ATP1A1

**Figure 5:**
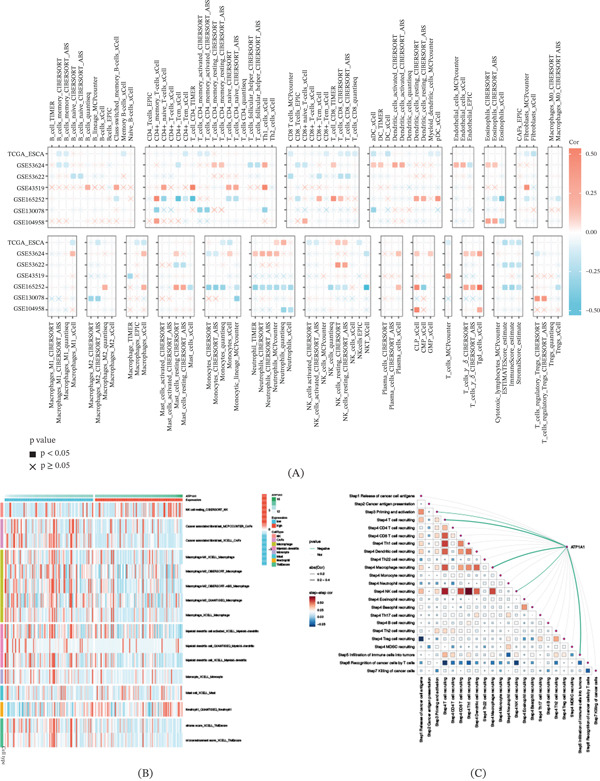
Association of ATP1A1 expression with immune infiltration and cancer immunity cycle activity. (A) Spearman’s correlation analysis between ATP1A1 expression and immune cell infiltration estimated by multiple algorithms. (B) Differences in immune cell infiltration between ATP1A1 high‐ and low‐expression groups based on seven independent deconvolution methods. (C) Correlation analysis between ATP1A1 expression and TIP cancer immunity cycle scores, together with correlation structure among TIP steps.

To further characterize the association between ATP1A1 expression and the immune microenvironment in esophageal cancer, multiple immune deconvolution algorithms were applied. Spearman’s correlation analysis demonstrated that ATP1A1 expression showed heterogeneous associations with different immune cell populations across datasets and computational methods, with both positive and negative correlations depending on the specific cell type and algorithm, suggesting that ATP1A1‐related immune features cannot be explained by a single estimation approach (Figure 5A). When tumors were stratified according to ATP1A1 expression levels, significant variations in the abundance of multiple immune‐related components were detected between ATP1A1 low‐ and high‐expression groups, including resting NK cells, cancer‐associated fibroblast‐related signatures, macrophage‐associated signatures, myeloid dendritic cells, monocytes, mast cells, neutrophils, and stromal and microenvironment scores, further supporting a relationship between ATP1A1 and immune composition remodeling in esophageal cancer (Figure 5B). Evaluation of cancer immunity cycle activity using TIP scores indicated that ATP1A1 expression was significantly associated with several steps of the cycle, and most significant associations were negative, suggesting that elevated ATP1A1 expression may be related to reduced activity in specific immune response processes rather than a uniform enhancement of antitumor immune function (Figure 5C). In addition, correlation analysis among TIP scores revealed substantial interstep connectivity, particularly among multiple immune cell recruitment–related steps, highlighting the coordinated organization of immunity cycle activity in the tumor microenvironment. Overall, these results suggest that ATP1A1 expression is linked to complex alterations in immune infiltration patterns and cancer immunity cycle activity in esophageal cancer.

### 3.6. ATP1A1 Expression Is Associated With Drug Response Patterns Across Pan‐Cancer Cell Lines

**Figure 6:**
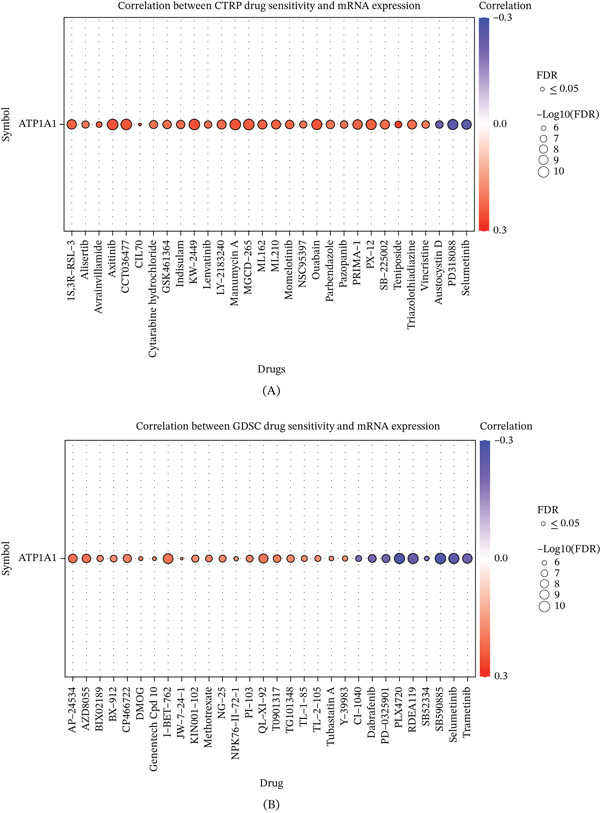
Association between ATP1A1 expression and drug sensitivity across pharmacogenomic datasets. (A) Correlation analysis between ATP1A1 expression and drug response in the CTRP dataset. (B) Correlation analysis between ATP1A1 expression and drug response in the GDSC dataset.

To explore the potential therapeutic relevance of ATP1A1 expression, pan‐cancer drug sensitivity analyses were performed using the CTRP and GDSC datasets. In the CTRP cohort, ATP1A1 expression was significantly associated with the sensitivity of multiple compounds, and most significant associations were positive correlations, indicating that higher ATP1A1 expression was generally linked to reduced drug sensitivity for a broad set of agents. Only a small subset of drugs showed negative correlations, suggesting increased sensitivity in ATP1A1‐high contexts for selected compounds (Figure 6A). In the GDSC cohort, ATP1A1 expression was also significantly associated with the response to multiple drugs; however, unlike the CTRP pattern, the correlations in GDSC showed a more mixed distribution, with both positive and negative associations observed across different agents (Figure 6B). Notably, several compounds exhibited negative correlations of relatively larger magnitude, indicating that elevated ATP1A1 expression may be linked to increased sensitivity to specific drugs in certain contexts. Together, these results suggest that ATP1A1 expression is broadly associated with heterogeneous drug response patterns across independent pharmacogenomic datasets, supporting its potential relevance to both drug resistance and drug vulnerability across cancer cell lines.

### 3.7. Single‐Cell Transcriptomic Localization of ATP1A1 Expression in Esophageal Cancer

**Figure 7:**
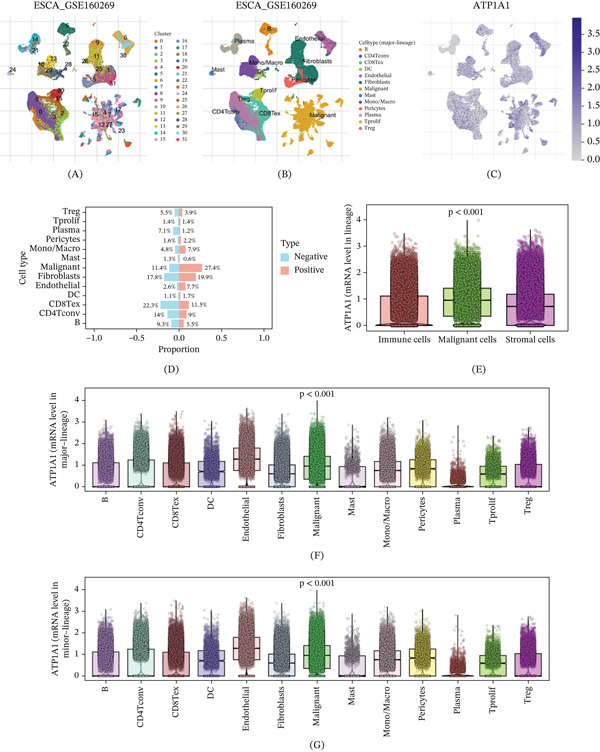
Single‐cell transcriptomic distribution of ATP1A1 expression in esophageal cancer. (A) UMAP visualization of single‐cell transcriptomes from the GSE160269 dataset. (B) Cell type annotation based on canonical marker genes. (C) Nebulosa density plot showing ATP1A1 expression distribution across cell populations. (D) Proportional distribution of cell types in ATP1A1‐positive and ATP1A1‐negative cells. (E–G) Comparison of ATP1A1 expression among different cell types using distribution plots and mean expression analysis. Statistical differences were assessed using the Kruskal–Wallis test.

Single‐cell RNA sequencing analysis of the GSE160269 esophageal cancer cohort was conducted to define the cellular distribution of ATP1A1 expression in the tumor microenvironment. UMAP analysis identified multiple cellular clusters that could be annotated as malignant cells, immune cell populations, and stromal components, outlining the major cellular architecture of esophageal cancer tissue (Figure 7A,B). Nebulosa‐based visualization showed that ATP1A1 expression was broadly distributed but unevenly enriched across the cellular landscape, with relatively stronger signals observed in the malignant cell region and in part of the stromal compartments, rather than being uniformly expressed across all cell types (Figure 7C). Comparison of ATP1A1‐negative and ATP1A1‐positive cells further demonstrated clear differences in cellular composition. ATP1A1‐positive cells were most prominently enriched in malignant cells and also contained substantial proportions of fibroblasts, CD8Tex cells, and Mono/Macro cells, whereas ATP1A1‐negative cells showed relatively higher proportions of B cells, CD4Tconv cells, plasma cells, and Tprolif cells (Figure 7D). At the lineage level, ATP1A1 expression differed significantly among immune, malignant, and stromal compartments, with malignant cells showing the highest overall expression level (*p* < 0.001; Figure 7E). More detailed comparison across annotated cell types further confirmed significant heterogeneity of ATP1A1 expression, with malignant cells exhibiting relatively high expression, while endothelial cells and some stromal or immune subsets also showed appreciable expression signals (Figure 7F,G). Overall, these results indicate that ATP1A1 is preferentially enriched in malignant cells at the single‐cell level, although its expression is not restricted to tumor cells alone.

### 3.8. Experimental Validation of ATP1A1 in Esophageal Cancer Cells

**Figure 8:**
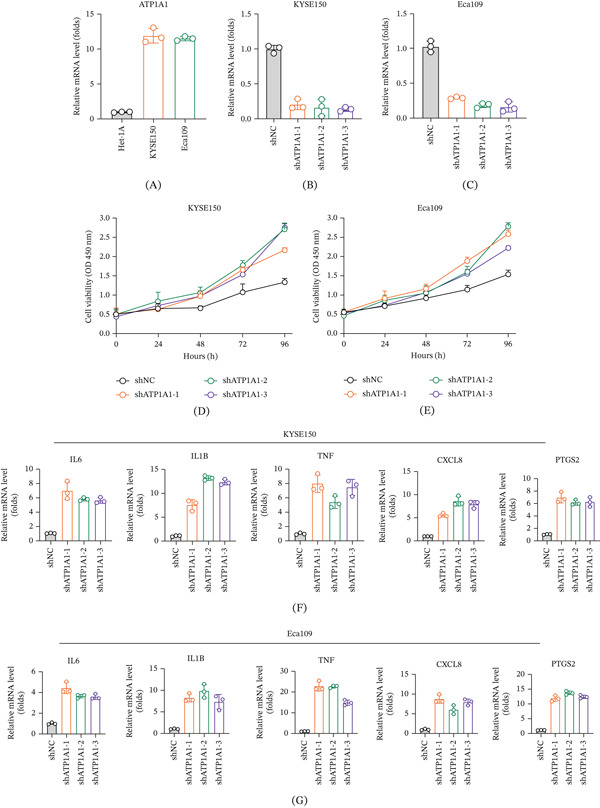
Experimental validation of ATP1A1 expression and function in esophageal cancer cells. (A) Relative ATP1A1 mRNA expression in normal esophageal epithelial cells (Het‐1A) and esophageal cancer cell lines (KYSE150 and Eca109). (B, C) Validation of ATP1A1 knockdown efficiency following transduction with three independent shRNAs. (D, E) Effects of ATP1A1 knockdown on cell proliferation assessed by CCK‐8 assays. (F, G) Relative expression of inflammatory genes (IL6, IL1B, TNF, CXCL8, and PTGS2) after ATP1A1 silencing. Expression levels were normalized to GAPDH. Data are shown as mean ± SD from three independent experiments.

To experimentally validate ATP1A1 expression in esophageal cancer cells, qPCR was first performed in the normal esophageal epithelial cell line Het‐1A and two esophageal cancer cell lines, KYSE150 and Eca109. ATP1A1 expression was markedly elevated in both KYSE150 and Eca109 cells compared with Het‐1A cells (Figure 8A), indicating aberrant upregulation in esophageal cancer cells. Stable ATP1A1 knockdown models were then established using three independent shRNAs, and qPCR confirmed that ATP1A1 expression was effectively reduced in both KYSE150 and Eca109 cells after shRNA transduction (Figure 8B,C). CCK‐8 assays further showed that ATP1A1 knockdown enhanced the proliferative capacity of both KYSE150 and Eca109 cells compared with shNC, particularly at later time points (Figure 8D,E), supporting a growth‐suppressive role of ATP1A1 in these cells. In addition, qPCR analysis demonstrated that ATP1A1 knockdown was accompanied by overall increased expression of multiple inflammatory genes, including IL6, IL1B, TNF, CXCL8, and PTGS2, in both KYSE150 and Eca109 cells (Figure 8F,G), although the magnitude of change varied among different shRNAs and between the two cell lines. Overall, these experimental findings support that ATP1A1 is highly expressed in esophageal cancer cells and that its depletion promotes cell proliferation, accompanied by elevated inflammatory gene expression.

## 4. Discussion

In this study, we performed an integrative transcriptomic analysis to investigate the prognostic and biological relevance of mitochondrial stress–related genes in esophageal cancer and ultimately prioritized ATP1A1 for in‐depth characterization. Although the LASSO model generated a five‐gene signature that provided cohort‐level risk stratification, ATP1A1 was selected as the focus of subsequent analyses because it was the only LASSO‐retained gene that also demonstrated consistent individual prognostic significance across both overall survival and disease‐specific survival analyses. This distinction is important because inclusion in a multigene prognostic model does not necessarily imply that each component gene has equally robust standalone relevance. By combining bulk RNA sequencing, immune profiling, single‐cell transcriptomics, and pharmacogenomic analyses, we further showed that ATP1A1 was associated with survival outcomes, metabolic transcriptional programs, immune‐related features, and drug response patterns. Single‐cell analysis additionally indicated that ATP1A1 expression was preferentially enriched in malignant epithelial cells, while remaining detectable in selected stromal and immune populations. Functional validation experiments further supported the biological relevance of ATP1A1 by showing that its knockdown promoted esophageal cancer cell proliferation.

Mitochondria are increasingly recognized as signaling organelles that integrate metabolic stress, redox balance, apoptosis, and inflammatory responses in cancer [37–39]. Dysregulation of mitochondrial stress pathways has been linked to tumor progression and therapeutic resistance across multiple malignancies [40–42]. However, the prognostic implications of mitochondrial stress–related genes in esophageal cancer have not been systematically explored. By applying LASSO Cox regression to a curated mitochondrial stress–related gene set, our study refines this pathway‐level concept by identifying ATP1A1 as a representative prognostic gene, highlighting its potential as a robust prognostic indicator rather than a diffuse pathway‐level signal.

ATP1A1 encodes the *α*1 subunit of the Na^+^/K^+^‐ATPase, a membrane protein traditionally known for maintaining ion gradients and cellular homeostasis [43]. Beyond this canonical role, accumulating evidence suggests that ATP1A1 also participates in signal transduction, oxidative stress regulation, and membrane‐associated signaling complexes that may influence cancer cell behavior [44–46]. Aberrant ATP1A1 expression has been reported in several malignancies and has been linked to tumor progression and therapeutic response [47–49]. In the present study, our findings extend these observations to esophageal cancer and suggest that ATP1A1 is primarily associated with metabolic transcriptional programs. In addition, higher ATP1A1 expression was accompanied by negative correlations with EMT, invasion, and quiescence, suggesting that ATP1A1 may reflect tumor biological states related primarily to metabolic regulation and reduced mesenchymal/invasive features. Consistent with this interpretation, ATP1A1 knockdown promoted proliferation in esophageal cancer cells, further supporting its association with tumor biological behavior.

A notable aspect of this study is the comprehensive characterization of immune‐related features associated with ATP1A1 expression. The tumor immune microenvironment plays a pivotal role in esophageal cancer progression and therapeutic response, particularly in the context of immunotherapy [50, 51]. We observed that ATP1A1 expression was closely linked to broad alterations in immunomodulatory gene expression, including immune stimulatory and inhibitory molecules, chemokines, and HLA genes. Multialgorithm immune infiltration analyses further revealed that ATP1A1 expression correlated with distinct immune cell composition patterns, suggesting associations with tumor immune contexture rather than isolated immune cell types. These findings suggest that ATP1A1 may influence tumor–immune interactions indirectly through tumor cell–intrinsic programs that co‐occur with immune variation within the tumor microenvironment.

Importantly, bulk immune associations can be confounded by cellular heterogeneity. To address this limitation, we leveraged single‐cell RNA sequencing data from the GSE160269 esophageal cancer cohort to localize ATP1A1 expression at cellular resolution. Our analysis showed that ATP1A1 expression was preferentially enriched in malignant epithelial cells but was also detectable in selected stromal and immune cell populations. This cellular distribution supports the interpretation that the bulk transcriptomic associations linked to ATP1A1 are likely driven mainly by tumor cell–associated transcriptional programs, while also reflecting contributions from the surrounding tumor microenvironment. Therefore, ATP1A1 should not be interpreted as a strictly tumor cell–specific marker but rather as a gene showing preferential enrichment in malignant cells within a heterogeneous cellular ecosystem [52, 53].

Beyond prognostic and immunological associations, we explored the potential therapeutic relevance of ATP1A1 using pan‐cancer drug sensitivity analyses. ATP1A1 expression was associated with differential sensitivity to multiple anticancer agents across independent pharmacogenomic datasets, indicating that ATP1A1 may be linked to drug resistance or vulnerability phenotypes. Because these analyses were performed across heterogeneous cancer cell lines, these findings should be interpreted as exploratory associations rather than cancer‐type–specific therapeutic effects. While these findings are exploratory and do not imply direct clinical applicability, they provide a rationale for further investigation into ATP1A1 as a biomarker of therapeutic response or as a modulator of treatment sensitivity, particularly in combination with immune‐based strategies.

Several limitations of this study should be noted. First, the analyses were mainly based on retrospective public datasets, and prospective validation in independent esophageal cancer cohorts is still warranted. Second, although our multiomics approach reveals robust associations, the functional experiments included in this study remain preliminary, and additional mechanistic studies will be needed to further elucidate the biological roles of ATP1A1. Finally, drug sensitivity analyses were conducted at the cell line level and may not fully capture in vivo tumor complexity. Despite these limitations, the reproducibility of findings across multiple analytical layers supports the robustness of the observed associations and their potential clinical relevance in esophageal cancer.

## 5. Conclusions

In summary, this study identifies ATP1A1 as a prognostically relevant factor associated with tumor biological features, immune microenvironment characteristics, and therapeutic response heterogeneity in esophageal cancer. Integrative transcriptomic, single‐cell, and experimental analyses suggest that ATP1A1 is preferentially enriched in malignant cells but not restricted to them, highlighting its potential biological and clinical relevance in a heterogeneous tumor ecosystem.

## Author Contributions

H.Y. performed the data analysis, conducted the bioinformatics investigations, and drafted the manuscript. L.H. conceived and supervised the study, contributed to data interpretation, and critically revised the manuscript.

## Funding

This study was supported by the Jiangsu Province Qinglan Project for Academic Leaders among Young and Middle‐Aged Scholars; the Jiangsu Provincial Education Science Fourteenth Five‐Year Plan Project (No. B/2023/02/112); and the Excellent Teaching Team Fund of the Qinglan Project at Yangzhou Polytechnic University.

## Disclosure

Both authors read and approved the final manuscript.

## Ethics Statement

The authors have nothing to report.

## Conflicts of Interest

The authors declare no conflicts of interest.

## Supporting information


**Supporting Information** Additional supporting information can be found online in the Supporting Information section. Supporting Information. Table S1: shRNA sequences used for ATP1A1 knockdown. The table lists the target sequences of shRNAs designed against ATP1A1 and the corresponding negative control (shNC).

## Data Availability

The datasets analyzed in this study are publicly available. Bulk RNA sequencing data and corresponding clinical information for esophageal cancer were obtained from The Cancer Genome Atlas (TCGA) database. Single‐cell RNA sequencing data were retrieved from the Gene Expression Omnibus (GEO) database under the accession number GSE160269. Drug sensitivity data were obtained from the Genomics of Drug Sensitivity in Cancer (GDSC) and Cancer Therapeutics Response Portal (CTRP) databases. All data used in this study are available from the corresponding public repositories.
